# Unraveling a Complex Case: Brucellosis Manifesting as Fever of Unknown Origin, Septic Ankle Arthritis, and Iliacus Abscess

**DOI:** 10.7759/cureus.74147

**Published:** 2024-11-21

**Authors:** Aya Odeh, Farah Alkhaled, Shatha Soudi

**Affiliations:** 1 Ophthalmology, The University of Jordan, Amman, JOR; 2 Pediatrics, The University of Jordan, Amman, JOR; 3 Internal Medicine, Al-Saudi Hospital, Amman, JOR

**Keywords:** brucellosis, fever of unknown origin, hemolytic anemia, iliacus abscess, infection, septic arthritis

## Abstract

Brucellosis is an infectious disease caused by bacteria of the genus *Brucella*, predominantly affecting livestock and humans through contact or consumption. It is a major public health challenge, particularly in developing countries. Symptoms can be mild to severe, making diagnosis difficult and often resulting in more chronic problems if those issues are not addressed. Our case report is about a patient with brucellosis who ended up having multiple organ involvement and focusing on the benefits of early diagnosis and management. Our case report discusses a 61-year-old Jordanian male patient with a significant medical history, including heart failure, diabetes, and hypertension, who was admitted for left ankle pain, redness, and swelling after a recent travel to Italy. His symptoms began four months prior with abdominal pain, fever, anemia, and persistent gastrointestinal issues, worsening despite oral antibiotics. He developed progressive thigh pain and was ultimately diagnosed with septic arthritis, experiencing ongoing fever and new anemia. Blood tests indicated elevated erythrocyte sedimentation rate (ESR), C-reactive protein (CRP), and leukocytosis levels, while cultures were negative. Imaging revealed fluid collections around the right iliac and gluteus minimus muscles. A diagnosis of brucellosis was confirmed through lab tests. Management included doxycycline 100 mg and rifampicin 600 mg as part of the treatment protocol, hydration support, pain relief, and imaging-guided drainage to address the infection and its complications. In conclusion, this case highlights the diverse manifestations of brucellosis, including atypical symptoms such as fever of unknown origin (FUO), septic arthritis, and iliacus abscess. Studies underscore the need to consider brucellosis in the differential diagnosis of FUO, especially in endemic regions. A multidisciplinary management approach and a high level of clinical suspicion are crucial for achieving optimal outcomes in patients with this complex infection.

## Introduction

Brucellosis: a global public health concern

Brucellosis is a zoonotic bacterial infection caused by *Brucella* species, primarily transmitted to humans through direct contact with infected animals or consumption of unpasteurized dairy products. This disease poses a significant public health concern, particularly in developing countries where livestock farming is prevalent [1]. The clinical presentation of brucellosis is highly variable, ranging from mild flu-like symptoms to severe systemic complications, which complicates diagnosis. Due to its nonspecific symptoms, brucellosis is often misdiagnosed, especially in non-endemic regions, making timely diagnosis challenging [2]. If left untreated, chronic complications can develop, leading to long-term health issues. The disease can affect multiple organs, with the skeletal system being most involved, particularly in *Brucella* spondylitis, seen in 10% to 85% of patients. Spinal involvement is prevalent, occurring in 54% of cases [3]. The diagnosis of brucellosis relies on clinical evaluation and laboratory tests such as blood cultures, serological assays, and molecular techniques. However, diagnostic challenges persist due to the nonspecific nature of the symptoms and the difficulty in obtaining suitable samples for testing [4]. Treatment typically involves a combination of antibiotics, although the emergence of antibiotic-resistant *Brucella* strains presents a growing challenge. Immunizing livestock, particularly in high-risk areas, offers promise in reducing transmission [5].

This case report presents a unique clinical course, highlighting the diagnostic difficulties and treatment outcomes of a patient diagnosed with brucellosis. By sharing this case, we aim to raise awareness among healthcare professionals about the disease's clinical presentation and emphasize the critical importance of early recognition and management to prevent severe complications.

## Case presentation

A 61-year-old Jordanian male patient with a significant medical history of heart failure (ejection fraction of 35%), diabetes mellitus (DM), hypertension (HTN), dyslipidemia, and glaucoma presented with left ankle pain, redness, heat, and swelling. He has a 35-pack-year smoking history and a past uncomplicated catheterization. Four months prior, the patient traveled to Italy and consumed unpasteurized cheese, where he first developed symptoms, including abdominal pain, black stool, constipation, fatigue, and a fever reaching 39°C. This fever persisted without improvement and was accompanied by rigors, chills, tremors, and fatigue. Prior to the fever onset, he had experienced symptoms of anemia. Despite treatment with oral antibiotics, including metronidazole, vancomycin, prizma, and teicoplanin, his condition showed no improvement.

**Figure 1:**
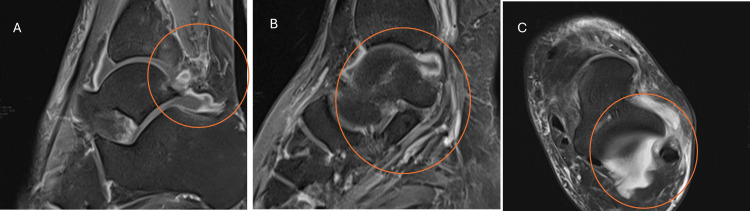
MRI of the left ankle indicating septic arthritis The magnetic resonance imaging (MRI) of the left ankle reveals a thick, peripherally enhancing effusion involving the talocrural and subtalar joints, with diffuse subcutaneous edema overlying the lateral malleolus. Panel A: Sagittal MRI of the ankle joint. This view shows the (hyperintense) signal in the joint cavity, suggesting joint effusion due to septic arthritis. Septic arthritis often causes an increase in synovial fluid, visible as bright areas on T2-weighted images. Panel B: Another sagittal view of the ankle with a closer focus on joint structures. Panel C: Axial MRI view of the ankle, providing a cross-sectional perspective. This view shows the distribution of effusion within the joint space and can reveal the spread of infection to the surrounding soft tissues.

As the disease progressed, the patient developed pain in the right anterior thigh and limp, which was not relieved by treatment with methocarbamol and paracetamol. The patient later developed similar pain in the left ankle, which was diagnosed as septic arthritis via magnetic resonance imaging (MRI) (Figure 1). He underwent drainage, and cytology of the synovial fluid revealed a leukocyte count of 5,200 × 10^6^/L, yet his fever persisted.

Diagnostic findings

Laboratory tests indicated significant inflammatory activity, with the erythrocyte sedimentation rate (ESR) elevated to 115 mm/hour and C-reactive protein (CRP) at 255 mg/L. The white blood cell (WBC) count was 12.7 x 10^9^/L, indicating leukocytosis, and the hemoglobin (Hb) was low at 9 g/dL, suggesting anemia. Urinalysis and urine cultures were unremarkable, as were blood and swab cultures, which failed to identify any bacterial pathogens (Table 1).

**Table 1:** Key laboratory results These results provide crucial insights into the patient's overall health, guiding further diagnostic evaluation and management strategies. Hb: hemoglobin; ESR: erythrocyte sedimentation rate; CRP: C-reactive protein; WBC: white blood cells; HbA1c (glycated hemoglobin); FIT (fecal immunochemical test); Cpk (creatine phosphokinase)

Lab Test	Result	Interpretation	Reference Range
Hb	78 g/L	Anemia is likely related to a chronic disease or infection.	138–172 g/L
ESR	115	Significantly elevated, indicating substantial inflammation.	0–15 mm/hour
CRP	255	Suggests acute inflammation or infection.	≤0.8–1.0 mg/dL
Sodium spot urine	94.20 mmol/L	Normal	40–220 mmol/L
Synovial fluid crystals	No crystals seen	Rules out crystal-induced arthritis.	No crystals seen
Synovial fluid WBC	5200x10^6^/L	Indicate inflammation or infection	<200 cells/µL
Creatinine	1.41 mg/dl	Mild elevation, possibly due to renal impairment or dehydration.	<1 mg/dL
CPK	0.68 ukat/L	Within the normal range, indicating no significant muscle damage.	10–120 µg/L
FIT	Positive	Suggests gastrointestinal bleeding.	Negative
HbA1c	7.50	Indicates poor diabetes control.	4–5.6
Sodium serum	129 mmol/L	Low, suggesting hyponatremia.	135–145 mEq/L
Blood film	Mild anisocytosis, mild thrombocytopenia	RBC morphology shows mild anisocytosis and polychromasia; WBCs indicate mild neutrophilia.	Normal appearance of cells. Normal white blood cell differential

**Figure 2:**
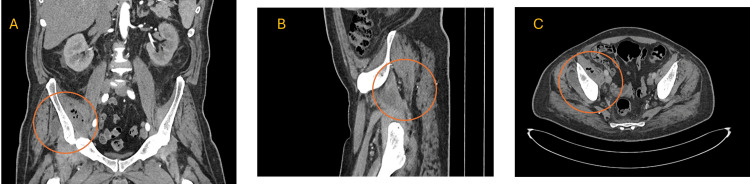
Computed tomography (CT) with IV contrast of the abdomen and pelvis An abdominal and pelvic CT with IV contrast showed large fluid collections with gas locules in the iliac and gluteus minimus muscles. Panel A: Coronal CT image of the abdomen and pelvis. The view shows hypodensity, representing fluid collection with internal gas locules deep to the right iliac and gluteus minimus muscles. Panel B: Abnormal findings in the sagittal CT image. Panel C: Axial CT image of the pelvis. This cross-sectional view provides a better look. The abnormalities include 29 x 44 x 88 mm fluid collections deep to the right iliac muscle and 22 x 38 mm to the right gluteus minimus muscle.

**Figure 3:**
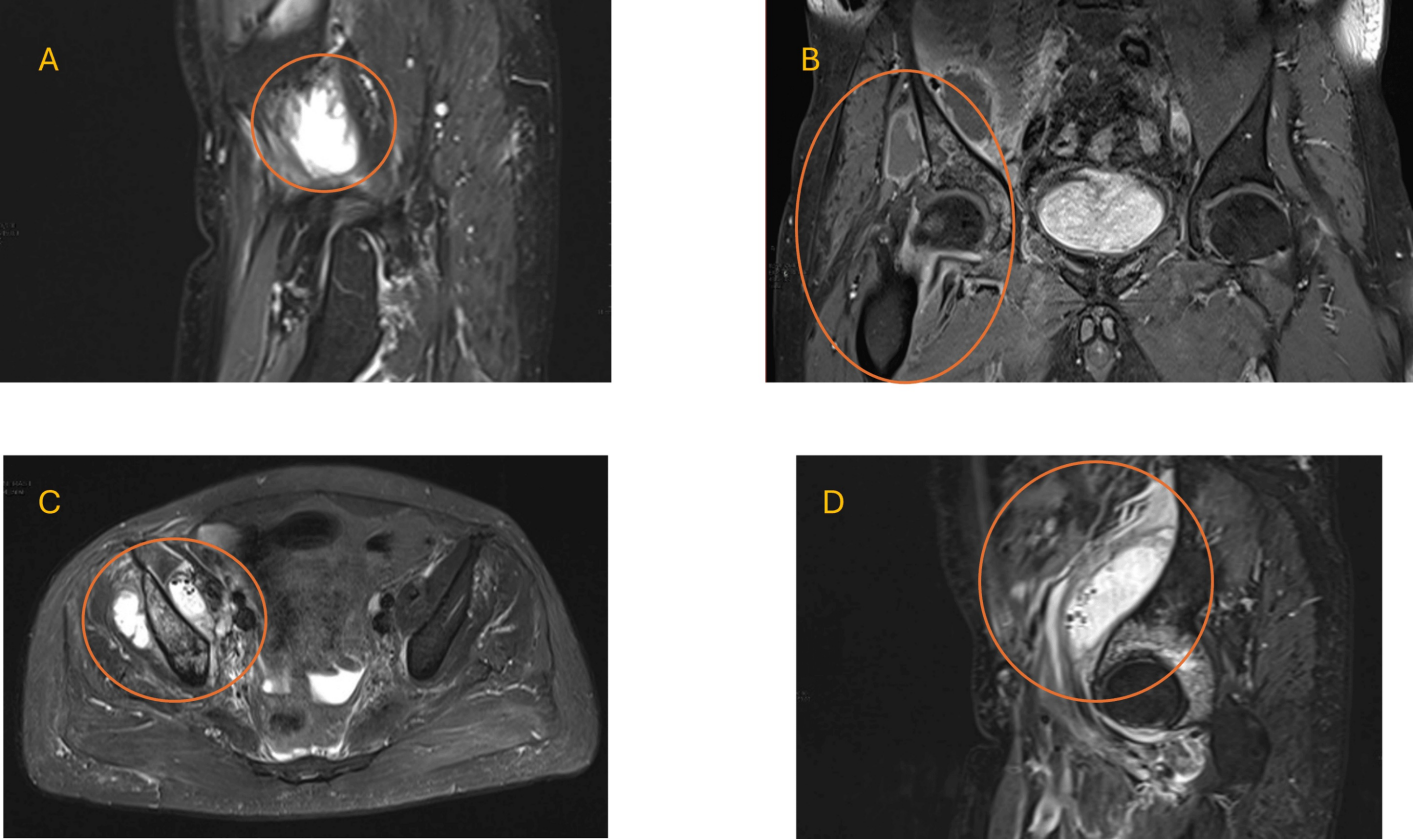
Abdomen and pelvis MRI with contrast. Pelvic MRI demonstrating collections in the iliac and gluteus minimus muscles with associated edema and reactive osteitis in adjacent bones. Panel A: Sagittal T2-weighted MRI showing the right iliac muscle, with ring-enhancing collection. The abnormality is hyperintense in the T2-weighted image. Panel B: Coronal T2-weighted MRI demonstrating a 3×5.8×8.6 cm ring-enhancing collection and a 4.8×3.8×3.3 cm with minimal edema involving the right iliac muscle and the right gluteus minimus. Note the irregular borders and heterogeneous signal intensity. Panel C: Axial T2-weighted MRI depicting ring-enhancing collection. The lesion is located in the right iliac and the right gluteus minimus muscles. Panel D: Another sagittal T2-weighted view highlighting an additional view of the abnormality noted in Panel A. The abnormal structure is seen in proximity to the right iliac muscle.

Multiple imaging studies were conducted to further investigate the patient's condition. X-rays of the chest, pelvis, and femur showed bilateral osteoarthritic changes in the pelvis and soft tissue calcification around the right hip joint. A CT scan of the chest, abdomen, and pelvis with IV contrast revealed significant fluid collections deep to the right iliac and gluteus minimus muscles. The largest fluid collection measured 29 x 44 x 88 mm with internal gas locules (Figure 2). Despite these findings, the patient continued to experience a fever of unknown origin (FUO), and imaging studies, including MRI, indicated ring-enhancing collections, diffuse muscle edema, and bone marrow edema suggestive of reactive osteitis and bursitis (Figure 3).

Management and diagnosis

The patient's management initially included intravenous antibiotics such as teicoplanin, piperacillin/tazobactam, and pain management with Perfalgan and pethidine. To prevent thromboembolic complications, enoxaparin and furosemide were administered. An ultrasound-guided drainage procedure was performed to manage the fluid collections, with thick, infected, yellowish fluid drained from the iliacus muscle abscess.

To rule out malignancy, endoscopic examinations were performed, yielding unremarkable findings except for hemorrhoids. Despite initial negative cultures, further testing for brucellosis was pursued due to the persistent FUO. The serological results confirmed the diagnosis of brucellosis with *Brucella abortus* 1/40, *Brucella melitensis* 1/80, and positive *Brucella* antibodies, confirming it as the underlying cause of his symptoms over the past four months.

Treatment

Upon confirming brucellosis, the patient was started on a six-week antibiotic regimen consisting of doxycycline (100 mg twice daily) and rifampicin (600 mg once daily). During this treatment period (six weeks), significant improvement in the patient's condition was observed, with the resolution of fever and progression toward recovery.

## Discussion

This case report focuses on a 61-year-old Jordanian male patient with multiple comorbidities. He presented with fever, abdominal pain, and joint symptoms following a trip to Italy. Initial symptoms began four months earlier, characterized by abdominal pain, melena, and persistent fever reaching up to 39°C, which did not improve with outpatient antibiotic treatment.

The patient developed left ankle pain and worsening right thigh pain. Laboratory tests showed elevated inflammatory markers, with an ESR of 115 mm/hour and CRP of 255 mg/L, indicating a significant inflammatory response. The WBC count was 12.7×10^9^/L, suggesting leukocytosis, while the hemoglobin level was low at 9 g/dL, indicating anemia. Urinalysis was normal, and cultures were negative, complicating the diagnosis.

In addition to laboratory tests, multiple imaging studies were performed, which identified significant fluid collections deep to the right iliacus and gluteus minimus muscles. Despite the persistent fever, serological tests for brucellosis eventually returned positive. Upon confirmation of the diagnosis, the patient was started on doxycycline (100 mg twice daily) and rifampicin (600 mg once daily) for a six-week duration.

This case underscores the importance of considering travel history and eating cheese in diagnosing infectious diseases and highlights the complexities involved in managing patients with multiple comorbidities, necessitating a multidisciplinary approach for optimal outcomes.

Moreover, this case illustrates a rare and complex manifestation of brucellosis, presenting as an FUO alongside complications such as septic ankle arthritis, anemia, and an iliacus abscess. These manifestations highlight the diagnostic challenges and the necessity of maintaining a high index of suspicion among clinicians, particularly in endemic regions.

Brucellosis overview

Brucellosis is a zoonotic infection primarily caused by the *Brucella* genus, transmitted from animals to humans, typically through unpasteurized dairy products or direct contact with infected animals.

The global incidence of brucellosis varies significantly, with higher prevalence observed in regions such as the Mediterranean, the Middle East, and parts of South Asia and Africa. In contrast, human brucellosis is rare in the United States, with only a few hundred cases reported annually, primarily among high-risk occupational groups, such as veterinarians and farmworkers [6].

*Brucella* species invade the reticuloendothelial system, effectively evading the host's immune response, which evade and undermine the innate and adaptive immune responses of the host through modulating the activation of pattern recognition receptors (PRRs), inflammatory responses, or the activation of immune cells like dendritic cells (DCs) to inhibit antigen presentation. Once in the bloodstream, these bacteria can disseminate to various organs, resulting in a wide range of clinical manifestations. Their ability to survive intracellularly contributes to both their pathogenicity and the chronic nature of the infection. Importantly, the presence of antibodies against *Brucella* does not always correlate with disease severity, complicating both diagnosis and treatment [7].

Brucellosis is characterized by a variety of symptoms, including fever, chills, malaise, and sweats, often leading to presentations of FUO. In our case, the patient exhibited FUO, septic ankle arthritis, and an iliacus abscess. These symptoms reflect the disease's nature and its potential to mimic other conditions, emphasizing the need for heightened clinical awareness.

Additionally, hemolytic anemia may occur due to immune-mediated mechanisms or direct invasion of red blood cells [8]. Septic arthritis and abscess formation arise from hematogenous spread, underscoring the critical importance of early intervention in managing brucellosis.

Diagnostic challenges

Diagnosing brucellosis is often challenging due to its nonspecific symptoms and variable presentations. Serological testing remains the cornerstone of diagnosis, with standard agglutination tests and enzyme-linked immunosorbent assays (ELISA) commonly employed. While culturing *Brucella* from blood or tissue samples is the definitive method, it can be time-consuming and may yield negative results, especially following prior antibiotic therapy. Imaging studies, such as ultrasound or MRI, may be necessary to identify abscesses or joint involvement.

Treatment of brucellosis typically involves a combination of antibiotics to enhance efficacy. The World Health Organization (WHO) recommends a regimen of doxycycline and rifampin for at least six weeks, with the addition of streptomycin or gentamicin for severe cases [9]. In this case, the early initiation of appropriate antibiotics was crucial in preventing complications. Surgical intervention may be required for abscess drainage or joint debridement in cases of septic arthritis.

Prognosis and prevention

The prognosis for brucellosis varies depending on the severity of the infection and the timeliness of treatment. Most patients respond well to appropriate antibiotic therapy; however, relapses can occur, particularly in severe or chronic cases [10]. Long-term complications may include spondylitis, chronic arthritis, or endocarditis. Our patient's prognosis improved significantly with prompt diagnosis and treatment, highlighting the critical importance of early recognition and intervention.

Preventive measures

Preventive measures are essential for controlling the spread of brucellosis. Public health strategies should focus on livestock vaccination, pasteurization of dairy products, and educating high-risk populations about safe handling practices for animals and animal products. Implementing robust surveillance systems can help monitor and control outbreaks, particularly in endemic areas [11]. Moreover, enhanced awareness among healthcare providers regarding the diverse presentations of brucellosis is vital for early diagnosis and the prevention of complications.

Brucellosis and FUO

The initial presentation of FUO poses a common challenge in clinical practice. Studies indicate that brucellosis can significantly contribute to FUO, with various reports demonstrating the prevalence of *Brucella* antibodies in affected patients. For instance, a study in Ethiopia found that 3.6% of patients with FUO tested positive for brucellosis, emphasizing the importance of including it in the differential diagnoses for FUO cases [12]. Similarly, research from China revealed that 54% of patients with confirmed brucellosis presented with FUO, particularly among individuals exposed to livestock [13].

These findings underscore the need to consider brucellosis when evaluating patients with unexplained fevers, especially in regions with known zoonotic transmission. In children, brucellosis has also emerged as a leading cause of FUO. A Turkish study demonstrated that 15.2% of children with FUO were diagnosed with brucellosis, advocating for increased awareness and prompt serological testing in pediatric populations presenting with FUO [14].

Septic arthritis

The development of septic ankle arthritis in our case aligns with existing literature, which highlights brucellosis's potential to cause significant joint involvement, including septic arthritis and osteoarthritis [15]. Early recognition and treatment are crucial to preventing joint damage and chronic complications. A report on septic arthritis caused by *Brucella melitensis* underscores the importance of maintaining a high suspicion for brucellosis in patients presenting with joint symptoms, particularly those with relevant exposure histories [16]. Timely identification of this condition can significantly improve patient outcomes, as demonstrated by successful interventions documented in similar cases.

Iliacus abscess

The iliacus abscess observed in our patient represents a rare but serious complication of brucellosis. Abscess formation can occur due to hematogenous spread or direct extension from adjacent infected tissues. The importance of imaging studies in diagnosing such complications cannot be overstated, as they enable early surgical intervention when necessary. This case contributes to the growing body of literature on the diverse complications of brucellosis and reinforces the need for thorough evaluation in cases of FUO.

One of the rarest types of abscesses is this one, where the abscesses form near the iliac muscle. Muscle abscesses due to brucellosis usually appear in the psoas and paravertebral muscles. The physical examination findings were normal, except for hip joint pain and limited range of motion. In the literature, only one case of a psoas abscess linked to brucellosis did not require surgical drainage and was effectively treated with anti-*Brucella* drugs. Psoas abscesses require surgical drainage and antibiotic therapy. In the only previous case in the literature, the abscess could not be drained because of its location, and it was not possible to obtain a specimen for culture. However, it was effectively treated with antimicrobials that are effective for brucellosis. MRI is an effective method for identifying spondylodiscitis, particularly in the initial phases, as well as paraspinal or epidural abscesses and nerve or root compression related to brucellosis. The diagnosis of both the iliacus muscle abscess and olecranon bursitis was made using MRI [17].

The bacteria can damage intervertebral discs and vertebral bodies, leading to cervical instability. Inflammatory granulation tissue and abscesses may compress the spinal cord, resulting in sensory and motor dysfunction in the limbs and potentially causing paraplegia. Pain is the most prevalent symptom among patients with brucellosis. While brucellosis-related spondylitis is uncommon, cervical spondylitis is particularly rare. This serious complication can lead to chronic pain, neurological issues, and paralysis if not accurately diagnosed and treated. Furthermore, the overuse of nonsteroidal anti-inflammatory drugs (NSAIDs) and antibiotics in China has made the typical fever pattern observed in brucellosis spondylitis patients less common, further complicating the diagnostic process [18].

Epidural abscesses most frequently occur in the lumbar vertebrae, while cervical spine involvement is relatively uncommon. The management of spinal epidural abscesses remains a topic of ongoing debate within the medical community. In certain cases, particularly among patients with stable neurological status, effective treatment has been achieved using antibiotics alone. However, when signs of spinal cord compression are present, the situation becomes a neurosurgical emergency due to the risk of rapid and progressive paralysis. This underscores the critical need for timely diagnosis and intervention to prevent severe neurological outcomes in patients affected by brucellosis-related complications.

Brucellosis can progress to systemic involvement, with the musculoskeletal system being especially susceptible to complications such as arthritis, bursitis, sacroiliitis, spondylitis, and osteomyelitis. Notably, a spinal epidural abscess is a rare but serious complication that may arise during spondylitis caused by *Brucella* species. While spine brucellosis is uncommon, it can be a debilitating manifestation of the disease [19].

Brucellosis can affect various internal organs, including the spleen, kidneys, para-aortic area, and even testicular and tubo-ovarian regions, leading to several genitourinary infections in males, such as brucella epididymal-orchitis (BEO), cystitis, prostatitis, interstitial nephritis, pyelonephritis, glomerulonephritis, and abscess formation in the kidneys and testes. BEO, although rare, occurs in approximately 5.7% of brucellosis cases and is usually unilateral. Symptoms of BEO include fever, scrotal pain and swelling, chills, malaise, fatigue, and headache. The incidence and severity of these complications can vary based on the specific *Brucella* strain, the patient's age, and the duration of the infection [20].

## Conclusions

This case study underscores the complexities of diagnosing and managing brucellosis, particularly in patients with multiple comorbidities. The patient, a 61-year-old man from Jordan, presented with atypical manifestations, including septic ankle arthritis and an iliacus abscess, which complicated the diagnostic process and delayed the initiation of appropriate treatment. Comprehensive diagnostic evaluations, including imaging studies such as CT scans and MRIs, were pivotal in identifying significant abscesses and joint involvement, which directly influenced treatment decisions such as surgical drainage. The reliance on serological testing, along with initial negative cultures, further delayed the diagnosis, emphasizing the diagnostic challenges posed by brucellosis, especially in non-endemic regions.

The patient responded well to a combination of antibiotic therapy and surgical drainage of abscesses, illustrating the importance of prompt treatment in preventing severe complications. This case contributes to the growing body of literature on brucellosis, highlighting how atypical findings like iliacus abscesses expand the clinical spectrum of the disease and emphasize the need for thorough evaluation in patients with unexplained symptoms. Overall, this case serves as a reminder of the persistent public health challenge posed by brucellosis and the necessity for ongoing education of healthcare professionals to effectively recognize and treat this zoonotic infection.
